# Structural and Immunological Features of PR-10 Allergens: Focusing on the Major Alder Pollen Allergen Aln g 1

**DOI:** 10.3390/ijms25094965

**Published:** 2024-05-02

**Authors:** Daria N. Melnikova, Ekaterina I. Finkina, Andrey E. Potapov, Yulia D. Danilova, Ilia Y. Toropygin, Natalia S. Matveevskaya, Tatiana V. Ovchinnikova, Ivan V. Bogdanov

**Affiliations:** 1 M.M. Shemyakin & Yu.A. Ovchinnikov Institute of Bioorganic Chemistry, Russian Academy of Sciences, 117997 Moscow, Russia; finkina@mail.ru (E.I.F.); cool.goyan@yandex.ru (A.E.P.); danilova-julia2307001@mail.ru (Y.D.D.); ovch@ibch.ru (T.V.O.); contraton@mail.ru (I.V.B.); 2 Institute of Biomedical Chemistry, 119121 Moscow, Russia; toropygin@rambler.ru; 3 G.N. Gabrichevsky Research Institute for Epidemiology and Microbiology, 125212 Moscow, Russia; matveevskaya@mail.ru

**Keywords:** allergy, sensitization, plant PR-10 proteins, pollen allergen, alder, Aln g 1, site-directed mutagenesis, lipid binding, IgE-binding capacity

## Abstract

Today, allergies have become a serious problem. PR-10 proteins are clinically relevant allergens that have the ability to bind hydrophobic ligands, which can significantly increase their allergenicity potential. It has been recently shown that not only the birch pollen allergen Bet v 1 but also the alder pollen allergen Aln g 1, might act as a true sensitizer of the immune system. The current investigation is aimed at the further study of the allergenic and structural features of Aln g 1. By using qPCR, we showed that Aln g 1 was able to upregulate alarmins in epithelial cells, playing an important role in sensitization. With the use of CD-spectroscopy and ELISA assays with the sera of allergic patients, we demonstrated that Aln g 1 did not completely restore its structure after thermal denaturation, which led to a decrease in its IgE-binding capacity. Using site-directed mutagenesis, we revealed that the replacement of two residues (Asp27 and Leu30) in the structure of Aln g 1 led to a decrease in its ability to bind to both IgE from sera of allergic patients and lipid ligands. The obtained data open a prospect for the development of hypoallergenic variants of the major alder allergen Aln g 1 for allergen-specific immunotherapy.

## 1. Introduction

**Figure 1:**
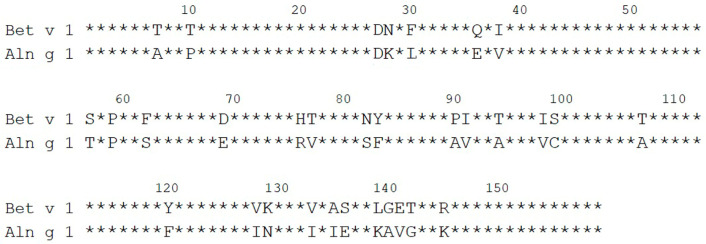
Alignment of aa sequences of Aln g 1 with Bet v 1.

Today, allergies are on the rise in Europe and are rated as chronic diseases affecting, based on the most conservative estimates, more than 60 million people [1]. The European Academy of Allergy and Clinical Immunology (EAACI) expects that by 2025, about one-half of the entire EU population may be taken ill with chronic allergic diseases [2]. In Europe and North America, plant panallergen PR-10 proteins represent a clinically relevant class of pollen and food allergens with a major member—the birch pollen allergen Bet v 1 [3]. PR-10 proteins include dozens of food and pollen allergen proteins with similar spatial structures across the plant kingdom, including Aln g 1 from *Alnus glutinosa* (alder) [3]. The major pollen allergen Aln g 1 is a clinically relevant allergen, as alder trees are widespread along with birch in North America and Central and North Europe regions. Previously, it was demonstrated that Bet v 1 is the primary sensitizer in allergies to the birch-related trees of the order Fagales and that the cross-reactivity of Bet v 1-specific IgE antibodies with its homologous pollen allergens (alder Aln g 1, hazel Cor a 1, etc.) triggers respiratory allergic symptoms beyond the birch pollen season [4]. It was shown that because of the high cross-reactivity of IgE, allergen-specific immunotherapy (AIT) with birch pollen extract is effective not only in patients with a birch pollen allergy but also in patients with allergy to pollen from such birch-related trees as alder, hazel, and oak [5,6]. But this is not the case for all patients. Based on T-cell reactivity, it was recently shown that alder Aln g 1, having a high degree of amino acid sequence similarity to Bet v 1 (88%) (Figure 1), may prove to be a genuine sensitizer of the immune system [4]. Previous IgE-competition ELISA experiments identified that Aln g 1 has a common cross-reactive with Bet v 1, as well as its own unique IgE-binding epitopes [4]. Independent sensitization of the immune system to the Aln g 1 allergen may explain why allergen-specific immunotherapy (AIT) with birch pollen extract or individual Bet v 1 is ineffective for some patients with PR-10-related allergies [4]. It was shown that specific IgE-blocking IgG developed during sublingual immunotherapy (SLIT) with the recombinant Bet v 1 (rBet v 1-SLIT) were cross-reactive with Aln g 1; however, they were unable to fully prevent Aln g 1-induced effector cell activation in the case of the majority of patients [7]. In addition, it is worth noting that plant panallergens of other classes, in particular, profilins and polcalcins, may be involved in sensitization and be responsible for the low effectiveness of AIT in some patients [8].

The key drawbacks of AIT are related to the risk of inducing local or systemic side effects in response to intact allergen administration. The impact of this drawback can be significantly reduced by the use of hypoallergenic variants of the allergens. There are several approaches to reduce the IgE-binding activity of the full-length allergen. The first approach includes the use of folding variants of the allergen: as most IgE-binding epitopes are conformational, disruption of spatial structure leads to a decrease in protein allergenicity. For example, clinical trials on humans (NCT00266526) demonstrated that the hypoallergenic denatured variant of Bet v 1 (rBet v 1-FV) had the same effectiveness as the intact allergen but caused less pronounced side effects [9,10]. The second approach involves the use of naturally occurring hypoallergenic isoforms of the allergen with reduced IgE-binding activity. For example, the Bet v 1d isoform, a natural component of birch pollen, reduces in vivo allergenicity and has been proposed as a promising candidate for use in immunotherapy of birch pollen-allergic individuals [11]. The third approach includes the introduction of amino acid substitutions into conformational B-cell epitopes of the allergen, which significantly reduces the ability of the allergen to bind with specific IgE. A number of amino acid residues have been identified for Bet v 1, which affect the IgE-binding capacity of the allergen [12,13]. For example, the substitution of conservative for homologs of Bet v 1 amino acid residues such as Glu45, Glu60, or Lys65 reduced the ability of Bet v 1 to bind to IgE from sera of allergic patients [12,13,14]. An important role in the IgE-binding capacity of birch pollen allergen was also shown for Asn28, Lys32, Pro59, Pro108, and Glu149 [12,15]. Despite the clinical significance, there are no such data for alder pollen allergen Aln g 1.

A significant feature of PR-10 allergens is the ability to bind lipids and other natural ligands because of the presence of hydrophobic cavities in their structure. It is known that plant pollen and foodstuffs contain a large number of hydrophobic molecules that can act as immune adjuvants and significantly increase the sensitization potential and allergenic properties of lipid-binding allergens [16]. For instance, it was shown that Bet v 1 penetrated through the epithelial barrier of allergic patients after binding with cholesterol via cholesterol- and glycolipid-rich caveolae [16]. It was previously shown that lipid molecules can impact the resistance of lipid-binding allergens to digestive enzymes [17] and lysosomal enzymes of antigen-presenting cells [18]. It has been shown that the preincubation of lipid-binding allergens with their ligands can increase the IgE-binding capacity of the allergens [17]. Therefore, the study of the ability of allergenic PR-proteins to bind ligands and the identification of key amino acid residues involved in ligand-binding may provide deeper molecular insight into their allergenicity and sensitization potential. In the case of Bet v 1, it was shown that Ile23, Leu24, Asp27, Lys54, and Tyr81 are involved in the binding of kinetins, Lys54 and Asp27 in the binding of sphingomyelin, and Asp27 in the binding of sphingosine [19]. As far as we know, there are no data about the lipid-binding capacity of Aln g 1 and its possible natural ligands.

The main goal of this study was to obtain new data on the allergenic and structural features of Aln g 1 in comparison with birch pollen allergen Bet v 1. Using gPCR, we investigated the ability of both pollen allergens to stimulate the expression of alarmin genes playing an important role in sensitization. Using fluorescence spectroscopy, we performed a comparative study of the ability of alder Aln g 1 and birch Bet v 1 to bind different hydrophobic ligands. Then, using CD-spectroscopy and ELISA assays, we studied the effects of heating on the structure of Aln g 1 and its ability to bind lipid ligands and IgE from sera of allergic patients. Moreover, we examined the influence of substitutions of such amino acids in the structure of Aln g 1 as Asp27 and Leu30, located at the entrance to the hydrophobic cavity of the allergen and possibly involved in the formation of protein–ligand complexes as well as in the interaction with specific IgE.

## 2. Results and Discussion

### 2.1. Influence on the Expression of Alarmin Genes in Epithelial Cells

**Figure 2:**
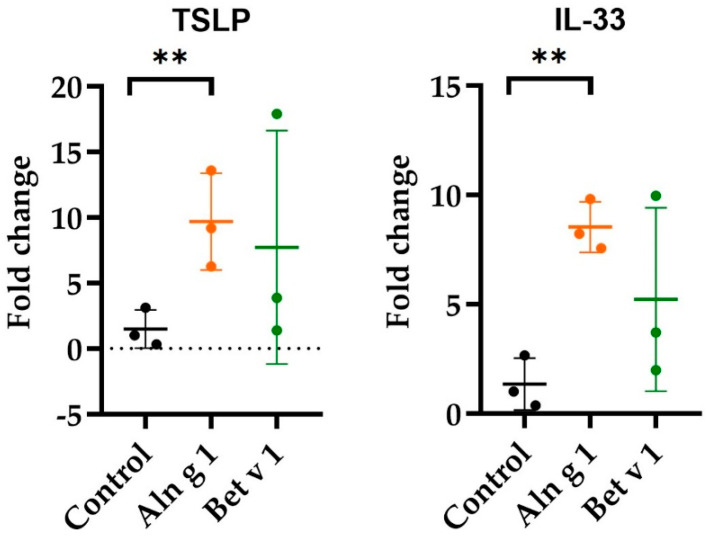
Analysis of relative gene expression levels of IL-33 and TSLP in Aln g 1- or Bet v 1-treated Calu-3 cells. Fold changes (2^−ΔΔCq^) are presented as mean ± SD. mRNA levels measured upon treatment were compared to the corresponding non-treated samples, and the graph was prepared using GraphPad Prism v 10.2.1. (GraphPad Software, San Diego, CA, USA). ** *p* ≤ 0.01.

It is currently known that epithelial cells act not only as a protective barrier but also as a sensor and integrator of environmental signals [20]. Earlier studies have revealed that the sensitization process may begin at the stage of allergen penetration through the respiratory epithelium, which leads to the production of alarmins TSLP, IL-33, and IL-25 by epithelial cells, triggering further allergic response of the immune system through innate lymphoid cells type 2 [16]. Previously, upregulation of TSLP, IL-33, and IL-25 was demonstrated for allergens of different classes, for example, peach Pru p 3, the major allergen of the class of lipid transfer proteins (LTPs) [21], kiwifruit cysteine protease Act d 1 [22], and peanut Ara h 3, belonging to the class of 11S globulins [23]. In this study, for the first time for homologs of Bet v 1, we examined the ability of birch Bet v 1 and alder Aln g 1 to affect the expression of alarmin genes in epithelial cells. As a part of this study, long-term exposure of the epithelium to Aln g 1 and Bet v 1 was carried out, which is typical for the pollination season. To do this, human airway epithelial cells (Calu-3) were stimulated for 24 h in a medium alone, as a control, or in a medium containing 5 μM of Aln g 1 or Bet v 1. It was demonstrated that Aln g 1 was able to stimulate the expression of TSLP and IL-33 genes (Figure 2). In the case of Bet v 1, the expression of TSLP and IL-33 varied quite strongly between one biological replication and the two other biological replications; however, it seemed that Bet v 1 was also able to induce the upregulation of TSLP and IL-33 genes. Because of the low representation of IL-25 transcripts, it was not possible to evaluate the expression of IL-25 in the case of both allergens. Based on this finding, we obtained data indicating the ability of the alder allergen Aln g 1 to cause sensitization of the immune system.

### 2.2. Effects of Heating on the Structure and Antibody-Binding Capacity of Alder Aln g 1

It is known that heating differently affects structures of various PR-10 allergens [24]. Denaturation of heat-labile allergens leads to the destruction of conformational epitopes and a decrease in the IgE-binding capacity of allergen molecules. Heat-denatured allergens not only bind IgE less effectively but also enhance Th1-dependent IgG2a. Therefore, heat-denatured allergens are suggested as hypoallergenic forms for more effective and safer AIT [25,26]. Here, we investigated the effect of heat treatment on the structure and IgE-binding capacity of alder Aln g 1 as compared to Bet v 1.

**Figure 3:**
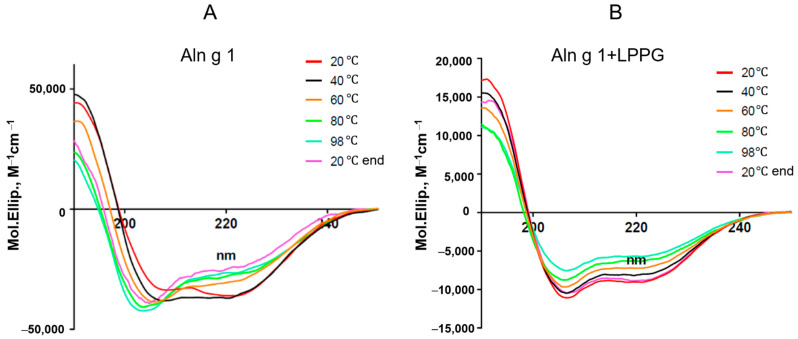
CD spectra of Aln g 1 upon heating without (**A**) and in the presence of the lysolipid LPPG (at the final concentration of 1 mM) (**B**). CD spectrum of the alder allergen at 20 °C after cooling down from 98 °C is designated as 20 °C end.

The effects of thermal denaturation on the secondary structure of Aln g 1 were investigated by CD spectroscopy (Figure 3, Table 1). The far-UV CD spectrum of Aln g 1 at pH 7.4 revealed a combination of α- and β-secondary structures characteristic for homologs of Bet v 1. The sample that was heat-treated up to 60 °C showed a slight shift in spectra, indicating the partial denaturation of Aln g 1. Heating up to 80 °C resulted in a further decrease in the relative content of the α-helical structure, but even at 98 °C, full allergen denaturation was not observed. At the same time, after cooling down to 20 °C, the CD spectrum of Aln g 1 did not take an original shape. These results were in contrast with our data that were previously obtained for soybean Gly m 4. At 98 °C, the soybean allergen protein unfolded, but after cooling down to 20 °C, the refolding of Gly m 4 took place [27]. However, as previously described, during heating, Bet v 1 underwent an unfolding process to a random coil structure and partly restored its structure after recooling [24]. The presence of lysolipid LPPG increased the percentage of α-helices in the Aln g 1 structure and provided greater resistance of the allergen to thermal denaturation, as described by us earlier for soybean Gly m 4 [27] (Figure 3, Table 1).

**Figure 4:**
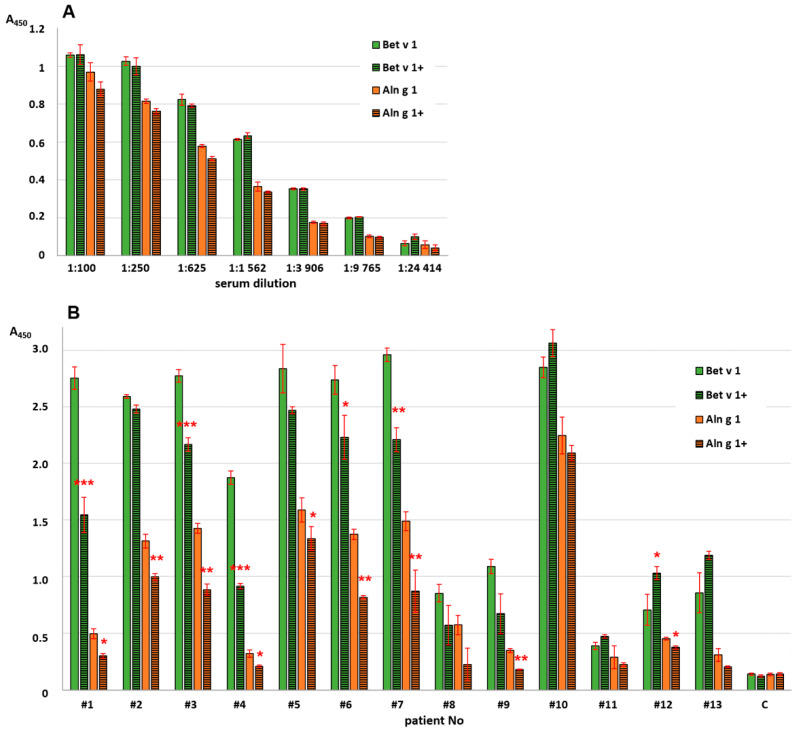
Antibody-binding capacity of intact (Bet v 1, Aln g 1) or preheated allergens (Bet v 1+, Aln g 1+): (**A**) interaction of allergens with polyclonal rabbit anti-Bet v 1 IgG and (**B**) interaction of allergens with IgE from sera of allergic patients. C—negative control (serum of non-allergic patients). Standard deviations between technical replications are shown as error bars. The *t*-test was used to compare A values between intact and preheated allergens (Bet v 1 or Aln g 1) for each patient (significance levels: * *p* < 0.05, ** *p* < 0.01, *** *p* < 0.005).

The effects of heating on the alder allergen capacity to bind human IgE from the sera of allergic patients as well as polyclonal rabbit anti-Bet v 1 IgG were examined by ELISA experiments (Figure 4, Supporting Materials, Table S1). Bet v 1 was used for comparison in these experiments. It was shown earlier that the polyclonal rabbit anti-Bet v 1 IgG not only effectively bound to Bet v 1 but also cross-reacted with Aln g 1 [27]. All selected sera from allergic patients contained sIgE capable of binding with birch Bet v 1 and, to a lesser extent, alder Aln g 1 (Figure 4, Supporting Materials, Table S1). Preheating the allergen for 20 min at 100 °C almost did not affect the rabbit IgG-binding capacity of either birch Bet v 1 or alder Aln g 1 (Figure 4). The same result was previously shown for food allergen Gly m 4 [27]. On the other hand, heating led to a significant decrease in the ability of both Bet v 1 and Aln g 1 to bind IgE from the sera of allergic patients (Figure 4). For some sera, this effect was more pronounced for Bet v 1. In the case of the other sera, heating had a stronger effect on the IgE-binding capacity of Aln g 1. Previously, for Mal d 1, which also does not restore its structure after heating, a decrease in IgE-binding capacity after incubation for 15 min at 80 °C and after 5 min at 100 °C was shown. Heat-treating Bet v 1 abolished its mediator-releasing capacity [24]. At the same time, for soybean Gly m 4, which, on the contrary, is capable of restoring its structure after heating, no obvious decrease in the efficiency of its interaction with IgE was observed [27]. Therefore, we concluded that upon heating, partial destruction of conformational epitopes in the Aln g 1 structure took place, which affected its ability to bind IgE from the sera of allergic patients, but not IgG from rabbit polyclonal antiserum.

Taking into account the CD-spectroscopy data and results of the ELISA assay, we assumed that the alder allergen Aln g 1 and birch Bet v 1 were not able completely to restore their structures after thermal denaturation. The conformational epitopes in the Aln g 1 structure were partially destroyed upon heating, which led to a decrease in its IgE-binding capacity. Therefore, heat-treated allergen Aln g 1 can be considered a hypoallergenic variant of the alder allergen.

### 2.3. Ligand-Binding Capacity of Aln g 1

It is well known that Bet v 1 and other PR-10 proteins are able to bind a wide range of different ligands, including flavonoids, fatty acids (FAs) and their derivatives, cytokinins, and other molecules, for example, sphingosine [19,28]. It was shown that flavonoid quercetin 3-O-sophoroside is a possible physiological ligand for Bet v 1 [29]. However, the assumption about the importance of this ligand for the IgE-binding capacity of the allergen and the development of allergic reactions has not yet been confirmed. It is interesting to note that phytosphingosine is a part of the natural ligand of the lipid-binding peach allergen Pru p 3 of the LTP class—10-OH-camptothecin-phytosphingosine—which determines its remarkable sensitizing capacity [30]. In a recent study, it was shown that the immunological activity of this ligand was determined by the phytosphingosine tail of 10-OH-camptothecin-phytosphingosine [31].

**Figure 5:**
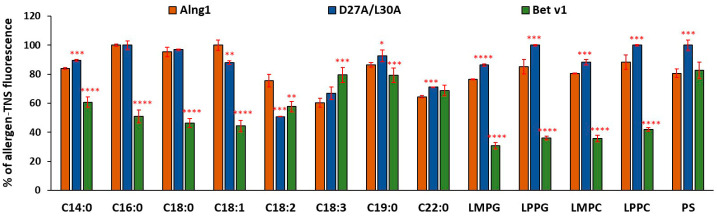
Effect of ligands on the fluorescence level of the protein–TNS complex. The results are expressed as the mean values (±SD) of the percentage of the fluorescence using the protein–TNS complex without ligand as a control. Data are representative of three experiments. Means with * (*p* < 0.05), ** (*p* < 0.01), *** (*p* < 0.005), and **** (*p* < 0.0001) are statistically significant compared with the fluorescence level of the Aln g 1-TNS complex. LMPC: 1-myristoyl-2-hydroxy-sn-glycero-3-phosphocholine; LMPG: 1-myristoyl-2-hydroxy-sn-glycero-3-phospho-(1′-rac-glycerol); LPPC: 1-palmitoyl-2-hydroxy-sn-glycero-3-phosphocholine; LPPG: 1-palmitoyl-2-hydroxy-sn-glycero-3-phospho-(1′-rac-glycerol); PS: phytosphingosine.

Here, using a fluorescent probe TNS, we first investigated the ability of Aln g 1 to bind various ligands in comparison with Bet v 1 (Figure 5). TNS is highly fluorescent when bound to a hydrophobic cavity of a protein. Based on the data from Bet v 1, the following groups of ligands were selected in this study: (a) FAs (12–18 carbon atoms) having acyl chains of different lengths and degrees of saturation, (b) lysolipids having different charge and chain lengths (14–16 carbon atoms), namely, lysophosphatidylcholine and lysophosphatidylglycerol, and (c) phytosphingosine. When lipid was added to a mixture of TNS and protein, the fluorescence of TNS became lower than that of the control (a mixture of proteins and TNS without lipids) equal to 100% (Figure 5). In contrast to Bet v 1, Aln g 1 bound tested lipids with much less in most cases. Among FAs, only C22:0 and C18:3 Aln g 1 bound with greater effectiveness than Bet v 1. In our experiments, TNS was more effectively displaced by all used lysolipids from the hydrophobic cavity of Bet v 1. At the same time, the alder allergen bound to phytosphingosine similarly to Bet v 1. It is worth noting that Aln g 1 and Bet v 1 completely lost this ability after heating.

Thus, we first showed the ability of alder Aln g 1 to bind various hydrophobic ligands. This allergen demonstrated less pronounced ligand-binding capacity than Bet v 1; the reason for this may be either structural features of the allergen or the existence of other ligands more specific for a given protein. It is worth noting that the spatial structure of Aln g 1 has not been established yet.

### 2.4. Effect of Amino Acid Substitutions on the Antibody- and Lipid-Binding Capacity of Aln g 1

**Figure 6:**
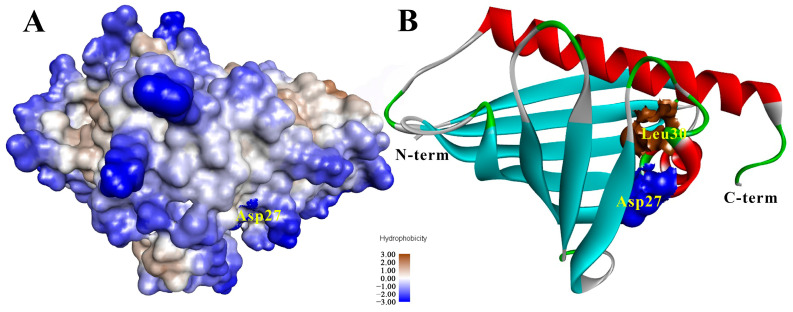
Predicted spatial structure of Aln g 1 in surface (**A**) and ribbon (**B**) representation. Spatial arrangement of Asp27 and Leu30. The structures were visualized in Discovery Studio Visualizer (Dassault Systèmes BIOVIA, Discovery Studio Visualizer, v20.1.0.19295, San Diego, CA, USA: Dassault Systèmes, 2020).

In the next stage of this study, we investigated the effect of amino acid substitutions on the structural and functional properties of the alder allergen. As noted above, the interaction of lipid-binding allergens of the PR-10 class with hydrophobic ligands, including those exhibiting immunogenic properties, can lead to an increase in their allergenic properties. Since we demonstrated that alder Aln g 1 is able to bind various hydrophobic ligands, we further aimed to test whether replacing the same residues could affect both the ligand-binding capacity of the allergen and its ability to interact with IgE from the sera of allergic patients. In most cases, single amino acid substitution does not significantly affect the properties of PR-10 proteins [32]. Taking this into account, we investigated the effect of replacing two amino acid residues, Asp27 and Leu30, in the structure of Aln g 1 located close to each other at the entrance to the hydrophobic cavity. Asp27, located on the surface of Aln g 1, is a negatively charged residue that is conserved for PR-10 allergens and is suggested to be involved in ligand binding of Bet v 1 (Figure 6) [19]. Contrarily, Leu30 is a hydrophobic residue located inside the protein cavity (Figure 6) and is a part of the dominant IgE-binding epitope of Bet v 1, comprising the region Bet v 129-58 [33].

A mutated variant of Aln g 1 D27A/L30A was obtained using site-directed mutagenesis. Analysis of the CD spectrum of the protein revealed that D27A/L30A had a structure similar to Aln g 1 consisting of a combination of α- and β-secondary structures (Supporting Materials, Figure S1). Based on previous findings that amino acid substitution in the structure of lipid-binding proteins of the LTP class could influence their structural characteristics and functional activity [28], we used computer simulations to investigate the size and volume of the hydrophobic cavity of D27A/L30A. It turned out that the introduced point mutations did not significantly affect the protein structure, but a slight increase in the volume of the hydrophobic cavity of Aln g 1 was observed: 1408 Å3 versus 1627 Å3 for the wild-type and double mutated analog D27A/L30A, respectively.

In the first stage of this study, we investigated the lipid-binding ability of D27A/L30A using the same spectrum as the tested ligands. It was shown that the introduced substitutions had different effects on the ability of the protein to form complexes with different types of ligands (Figure 6). D27A/L30A bound most FAs with similar or lower efficiency than Aln g 1 with the exception of C18:1 and C18:2. D27A/L30A bound these FAs better than the wild-type protein. At the same time, the mutant analog bound all lysolipids worse than Aln g 1, and in some cases, binding did not occur at all. The substitution of Asp27 and Leu30 in the Aln g 1 structure also led to the loss of the ability of the alder allergen to bind phytosphingosine. It was previously shown for Bet v 1 that the positively charged ammonium group of sphingosine is hydrogen bonded to Asp27 in the P-tunnel region [19]. It is possible that Asp27 in the structure of Aln g 1 plays a similar role, ensuring the formation of a protein complex with phytosphingosine, also containing an amino group. Summing up all the data obtained, we concluded that the simultaneous substitution of Asp27 and Leu30, which are both located at the entrance to the hydrophobic cavity, leads to a decrease in the ability of the alder allergen to bind hydrophobic molecules of different chemical structures. Since one of them is charged and located on the surface of the protein structure and the other is hydrophobic and located inside the cavity, the reason for the decrease in ligand-binding capacity may be the changes in the efficiency of both initiating interactions with the ligand and the ability of the protein to retain the ligand within the hydrophobic cavity.

**Figure 7:**
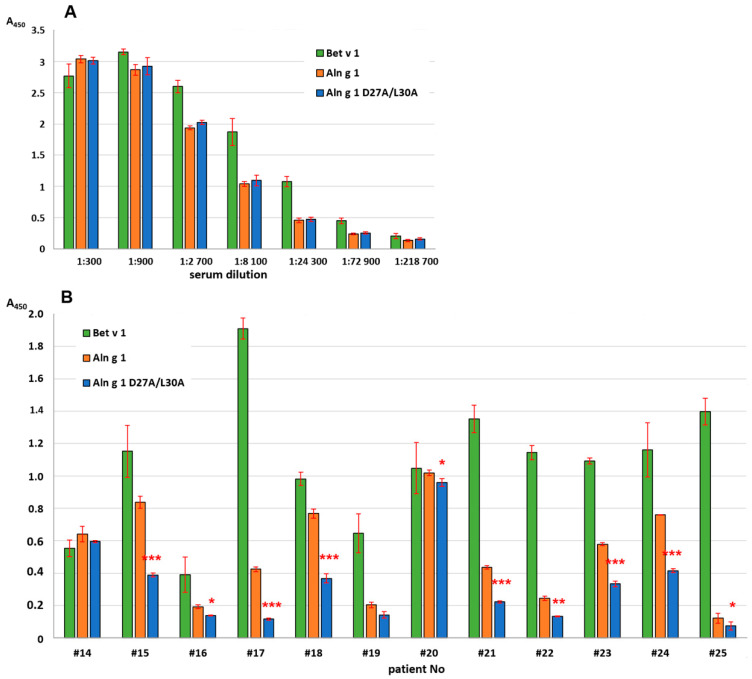
Antibody-binding capacity of Aln g 1 and its mutant analog D27A/L30A with IgE from the sera of allergic patients: (**A**) interaction of proteins with polyclonal rabbit anti-Bet v 1 IgG and (**B**) interaction of proteins with IgE from the sera of allergic patients. Data for Bet v 1 are given for comparison. Standard deviations between technical replications are shown as error bars. The *t*-test was used to compare A values between Aln g 1 and its mutant analog D27A/L30A for each patient (significance levels: * *p* < 0.05, ** *p* < 0.01, *** *p* < 0.005).

The next step was to study the possible role of the amino acid residues Asp27 and Leu30 in binding with antibodies. Rabbit anti-Bet v 1 antibodies [27] were used in the ELISA assay in order to compare the immunological properties of Aln g 1 and its mutant D27A/L30A. It was shown that polyclonal rabbit anti-Bet v 1 IgG cross-reacting with Aln g 1 bound the alder allergen and its mutant analog with equal effectiveness and replacement of the amino acid residues did not affect the interaction of the allergen with these antibodies (Figure 4). Another result was obtained in the IgE-binding experiments performed with sera from twelve allergic patients containing sIgE to Aln g 1 (Figure 7). A decrease in IgE-binding capacity of the D27A/L30A analog in the case of most sera was shown (Figure 7). Taking into account the lack of information on the B-cell epitopes of Aln g 1, we hypothesized that the replacement of the two residues located close to each other leads to the disruption of one of the conformational epitopes of the alder allergen responsible for IgE-binding.

It is important to note that the T cell-activating regions of Aln g 1 remained untouched in the D27A/L30A mutant: the main T cell epitopes of Aln g 1 involve regions Aln g 1112–123 and Aln g 1142–153 [7]. It is known that retaining T cell reactivity can induce an allergen-specific IgG-based immune response instead of an allergic IgE-based one, which leads to immunological tolerance [34]. Thus, here, we showed for the first time that the substitution of the two amino acid residues Asp27 and Leu30 in the structure of Aln g 1 leads to a simultaneous decrease in the ability of the alder allergen to bind both hydrophobic ligands and IgE from the sera of allergic patients. We suppose that simultaneously targeting both a decrease in IgE-binding and a reduction of lipid-binding may prove to be a good strategy for the development of allergen vaccines for PR-10 allergens in the future.

## 3. Materials and Methods

### 3.1. Materials

In all experiments, sera from patients (n = 62) with pollen and pollen-food allergies from the Moscow region with a higher prevalence allergy to birch were used (collected and kindly provided by the Clinical Diagnostic Center of the G.N. Gabrichevsky Research Institute for Epidemiology and Microbiology). RIDA qLine Allergy Panel 1–4 (R-Biopharm, Pfungstadt, Germany) was used to determine the amount of specific IgE (sIgE) to allergen extracts in the patient sera. The presence of sIgE to Aln g 1 in the sera of allergic patients was revealed in ELISA assays. The sera of 25 allergic patients containing sIgE to Aln g 1 were used in IgE-binding experiments with preheated allergens and the mutant of Aln g 1 D27A/L30A (Supporting Materials, Table S1). Recombinant Bet v 1 was used for comparison in all experiments. Sera samples from non-allergic individuals were used as a negative control.

### 3.2. Stimulation of Calu-3 Cells with Aln g 1 and Bet v 1

Human epithelial lung adenocarcinoma (Calu-3) cells (ATCC HTB-55) were cultured in Dulbecco’s Modified Eagle Medium/F12 (DMEM/F12) 50/50 mix with glutagro (Corning, Manassas, VA, USA), supplemented with 10% fetal bovine serum (FBS, Capricorn Scientific, Germany) and 1x antibiotic–antimycotic solution (Gibco, Thermo Fisher Scientific, Waltham, MA, USA). The cells were seeded in the wells of a 24-well plate at a density of 2.6 × 105 cells/cm^2^ and cultured in a humidified CO_2_ incubator (5% CO_2_, 37 °C), changing the medium twice a week until reaching a monolayer. One week later, the medium was replaced with a fresh medium without FBS. Then, 24 h later, the cells were treated with 5 μM of Aln g 1 or Bet v 1 in serum-free DMEM/F12 for 24 h. Medium alone without allergens was used as a control. Finally, 24 h later, the cells were lysed with ExtractRNA reagent (Evrogen, Moscow, Russia) and frozen at −70 °C.

### 3.3. RNA Extraction

Total RNA was extracted from the frozen samples according to the manufacturer’s instructions using ExtractRNA reagent. Three independent biological replicates for each condition were extracted on different days. The assessment of RNA integrity was performed by agarose gel electrophoresis, and the quantity and quality of isolated RNAs were estimated with a UV/Vis spectrophotometer on a Nanophotometer NP50 (Implen Inc., Westlake Village, CA, USA) (OD260/280 values for all isolated RNA samples were above 1.8).

### 3.4. Real-Time PCR

For each biological replication, 2 μg of total RNA was reverse-transcribed using an MMLV kit (Eurogen, Chania, Greece) according to manufacturer’s instructions. Then, 40 ng of cDNA synthesis was used in qPCR experiments using qPCRmix-HS SYBR (Eurogen) and 1 μM of each gene-specific primer in a final volume of 10 μL. qPCR reactions were carried out on a CFX Opus 96 (Bio-Rad, Hercules, CA, USA). The following thermal cycle profile for PCR was used: 95 °C—3 min to activate the hot-start DNA polymerase, followed by 50 cycles of 95 °C—15 s, 62 °C—20 s, and 72 °C—30 s. Each reaction was performed in technical duplicates, and the mean of three independent biological replicates was calculated. For each gene, primer efficiency was assessed using the RDML-LinRegPCR online tool [https://www.gear-genomics.com/rdml-tools/linregpcr.html, accessed on 1 March 2024], and efficiency between 85 and 105% was obtained. For analysis of qPCR data, a 2^−ΔΔCq^ method was used [35].

### 3.5. Production of Aln g 1 and It Mutant

Aln g 1 was produced as described earlier, with minor modifications [7]. The expression plasmids pET-His8-Aln g 1 and pET-His8-pET-His8-Aln g 1(D27A/L30A) was obtained by ligating the EcoRI/BamHI fragment of original or mutant genes into the expression vector pE2-His8, which encodes for an 8-residue His-Tag fusion protein. The mutant gene was obtained by site-directed mutagenesis of the original gene using PCR amplification with mutagenizing primers (Supporting Materials, Table S2). Plasmids containing the DNA insert of Aln g 1 or its mutant were overexpressed in E. coli. Protein expression was induced using 0.1 mM of isopropyl-D-1-thiogalactopyranoside for 4 h at 37 °C. Purification of the recombinant proteins was carried out by using consecutive IMAC, dialysis, and RP-HPLC. Homogeneity and the identity of the recombinant allergens were confirmed by SDS-PAGE.

### 3.6. CD Spectroscopy

Circular dichroism spectra of Aln g 1 and its mutant in 10 mM phosphate buffer, pH 7.4, were recorded at different temperatures as described in [17]. LPPG at a final concentration of 1 mM was used in these experiments.

### 3.7. Immunoglobulin Binding Assay

ELISA assays with the polyclonal anti-Bet v 1 rabbit antiserum were performed as described in [27]. Briefly, the recombinant allergens (0.5 μg/well) in phosphate-buffered saline (PBS), pH 7.4, were adsorbed into the plate wells. Serial dilutions of anti-Bet v 1 rabbit antiserum in PBS with 0.5% BSA were added to the plate wells and incubated overnight at 4 °C. The peroxidase-conjugated anti-rabbit IgG in PBS with 0.5% BSA and TMB liquid substrate was used for detection. To study the binding of IgE from the sera of allergic patients with recombinant allergens, ELISA assays were performed in a similar way [27]. For that, the sera of allergic patients in 1:4 dilution in PBS with 0.5% BSA were added to the plate wells and incubated overnight at 4 °C. IgE-binding was detected by using the peroxidase-conjugated anti-human IgE and TMB liquid substrate, supersensitive for ELISA (Merck Millipore, Darmstadt, Germany). For the IgE-binding experiments with intact Aln g 1 and Bet v 1 and Aln g 1 and Bet v 1 heated at 100 °C for 20 min, the sera of thirteen patients containing sIgE to Aln g 1 were used. For the comparison of the IgE-binding capacity of Aln g 1 and its mutant D27A/L30A, the sera of another twelve patients containing sIgE to Aln g 1 were used because of the limited amount of biological material. Each experiment was carried out twice.

### 3.8. Computational Studies

For computational studies, the 3D model of Aln g 1 predicted earlier and deposited into the AlphaFold Protein Structure Database [ID P38948] was used. Mutations D27A/L30A were introduced into the 3D structure of Aln g 1 using PyMOL 1.8.2.0 software (Schrodinger, LLC, New York, NY, USA) [24]. The volumes of Aln g 1 and D27A/L30A hydrophobic pockets were measured using the CASTp 3.0 tool (http://sts.bioe.uic.edu/castp, accessed on 1 March 2024) with a 0.56 Å probe radius [36].

### 3.9. Ligand Binding

The ability of Bet v 1, Aln g 1, and its mutant (D27A/L30A) to bind ligands was assayed by monitoring the displacement of the fluorescent, hydrophobic probe 2-p-toluidinonaphthalene-6-sulphonate (TNS). Ligand-binding experiments were performed at 25 °C in an F-2710 spectrofluorimeter (Hitachi High Technologies America Inc., Pleasanton, CA, USA), as described previously [37] with minor modifications. The excitation wavelength was set at 320 nm and the emission wavelength was scanned between 330 and 550 nm with a scan speed of 1500 nm/min. TNS, with or without the tested ligands, was incubated for 1 min in a stirred cuvette containing 2 mL of the buffer (175 mM mannitol, 0.5 mM K_2_SO_4_, 0.5 mM CaCl_2_, 5 mM MES (pH 7.2)) before the initial fluorescence (F0) was recorded. The results were expressed as a percentage of the protein–TNS complex fluorescence calculated using the formula [(F − F0)/FC] × 100%, where FC is the fluorescence of the allergen–TNS complex in the absence of a ligand. Each experiment was performed in triplicate, independently. Data were expressed as means ± SDs, which were calculated in all treatments using GraphPad Prism. Significant differences between means were analyzed by the *t*-test.

## 4. Conclusions

Aln g 1 is a clinically relevant but under-investigated allergen from alder pollen. This allergen takes part in the development of allergic cross-reactions with the major allergen of the PR-10 class—the birch pollen Bet v 1. However, it has recently been shown that Aln g 1 is able to cause Bet v 1-independent sensitization of the immune system, which might be a reason for the ineffectiveness of AIT carried out with Bet v 1. In this study, we obtained new data regarding the structural and allergenic features of Aln g 1. Using qRCR experiments, we showed that Aln g 1 was able to stimulate the expression of alarmin genes in epithelial cells that play an important role at the initial stage of sensitization. Using fluorescence spectroscopy, we demonstrated that Aln g 1 bound various hydrophobic ligands with different effectiveness. Based on the CD-spectroscopy data and the ELISA assay, we assumed that the alder allergen Aln g 1, as well as Bet v 1, did not completely restore its structure after thermal denaturation, which led to the partial destruction of conformational epitopes of the allergen and decrease in its IgE-binding capacity. Using site-directed mutagenesis, we demonstrated that replacing the same residues in the molecule of Aln g 1 simultaneously affected the ligand- and IgE-binding capacity of the allergen. We revealed that the substitution of two residues, Asp27 and Leu30, located at the entrance to the hydrophobic cavity of Aln g 1, on the one hand, might lead to disruption of one of the conformational epitopes of the allergen and thereby to a decrease in its ability to bind IgE from sera of allergic patients. On the other hand, the replacement of these residues might lead to a change in the structural organization of the allergen and a decrease in its ability to form complexes with various lipid ligands. Our data suggest that heat-treated Aln g 1 and its mutant D27A/L30A can be considered as possible hypoallergenic variants of the alder allergen for safer AIT. However, further in vivo experiments using animal models of allergic sensitization are required.

## Figures and Tables

**Table 1:** Secondary structure estimation (%) predicted from far-UV CD spectra.

Sample	Conditions	α-helix, %	β-sheet, %	β-turn, %	Random, %	NRMSD *
Aln g 1	20 °C	14.0	33.6	20.4	32.1	0.02
	40 °C	13.8	33.1	21.1	32.0	0.02
	60 °C	10.2	35.9	21.9	32.0	0.02
	80 °C	9.4	35.6	22.5	32.6	0.02
	98 °C	8.8	35.6	22.5	33.0	0.04
	20 °C end	9.6	34.6	21.5	34.4	0.03
Aln g 1 +LPPG	20 °C	32.6	18.9	21.4	27.1	0.03
	40 °C	32.6	18.6	21.4	28.4	0.04
	60 °C	33.3	23.7	17.7	25.2	0.07
	80 °C	21.5	25.2	22.0	31.3	0.03
	98 °C	21.1	26.2	22.1	30.6	0.02
	20 °C end	29.0	19.7	21.8	29.6	0.02

* The NRMSD is a fit parameter, which is a measure of the difference between the experimental ellipticities and the ellipticities of the back-calculated spectra for the derived structure. The optimal value of NRMSD should be <0.1.

## Data Availability

Data is contained within the article (and Supplementary Materials).

## Supplementary Materials

The following supporting information can be downloaded at: https://www.mdpi.com/article/10.3390/ijms25094965/s1.
