# Supplementary material for: Structural and Immunological Features of PR-10 Allergens: Focusing on the Major Alder Pollen Allergen Aln g 1
Source: Int J Mol Sci. 2024 May 2;25(9):4965. doi: 10.3390/ijms25094965 (PMC11084660; doi:10.3390/ijms25094965)
Supplement: Supplementary file 1 [file ijms-25-04965-s001.zip › ijms-2966821-supplementary.pdf]

Supporting Materials

Structural and immunological features of PR-10 allergens: the focus on major alder pollen allergen Aln g 1

Daria N. Melnikova[1]*, Ekaterina I. Finkina[1], Andrey E. Potapov[1], Yulia D. Danilova[1], Ilia Y. Toropygin[2], Natalia S. Matveevskaya[3], Tatiana V. Ovchinnikova[1], Ivan V. Bogdanov[1]

1 M.M. Shemyakin & Yu.A. Ovchinnikov Institute of Bioorganic Chemistry, the Russian Academy of Sciences, 117997 Moscow, Russia, contraton@mail.ru (I.V.B.), cool.goyan@yandex.ru (A.E.P.), finkina@mail.ru (E.I.F.); danilova-julia2307001@mail.ru (Y.D.D.), ovch@ibch.ru (T.V.O.)
2 Institute of Biomedical Chemistry, 119121 Moscow, Russia; toropygin@rambler.ru (I.Y.T.)
3. G.N. Gabrichevsky Research Institute for Epidemiology and Microbiology, 125212 Moscow, Russia, matveevskaya@mail.ru (N.S.M.)
* Correspondence: d_n_m_@mail.ru (D.N.M.).

Table S1. Characterization of patient sera containing sIgE to Aln g 1.

No.	Age (y.o.)	Sex (M/F)	sIgE to birch		sIgE to alder		Another allergen source
			IU/ml	RAST class	IU/ml	RAST class	
1	4	M	>100	6	>100	6	H, G, ct, wt, hz, p, po, wn, am, s
2	4	M	>100	6	55.12	5.1	H, G
3	6	M	>100	6	8.95	3.3	H, hz
4	5	M	>100	6	>100	6	H, G, O
5	5	M	75.31	5.5	14.15	3.7	H, G, p, wh, ct, hz, s
6	2	M	>100	6	>100	6	H
7	7	M	45.95	4.8	7.92	3.3	H
8	4	M	12.86	3.6	0.73	2	H, G
9	3	M	>100	6	>100	6	H, G, O, hz, ct, wh, cl, ap, wn, am, po, s
10	7	M	>100	6	9.2	3.4	H, hz
11	6	F	40 .64	4.7	9.7	3.4	H
12	6	M	>100	6	85.39	5.7	H, G
13	9	M	90.88	5.8	16.74	3.9	H, G, hz
14	8	M	>100	6	>100	6	H, hz, ct, po

15	6	M	>100	6	nd	nd	ct, ap
16	6	M	>100	6	>100	6	hz
17	6	F	>100	6	nd	nd	ct, ap
18	12	F	>100	6	>100	6	H, O, wn, cl, ap, wh, r
19	5	F	>100	6	16.78	3.9	H
20	68	M	>100	6	24.91	4.2	H, O
21	4	M	>100	6	17.37	3.9	H
22	16	F	>100	6	17.18	3.9	H, O, hz
23	25	M	>100	6	15.82	3.8	H
24	4	M	21.46	4.1	nd	nd	s, hz, p, ct
25	11	F	>100	6	nd	nd	s, hz

nd – not determined

Description of RAST classes: 0 class – not detectable or trace IgE level [0.00-0.34 IU/ml]; 1 class – threshold level [0.35-0.69 IU/ml]; 2 class – elevated IgE level [0.70-3.49 IU/ml]; 3 class - significantly elevated IgE level [3.50-17.49 IU/ml]; 4 class – high IgE level [17.5-49.99 IU/ml]; 5 class – very high IgE level [50.0-99.99 IU/ml]; 6 class - extremely high IgE level [⩾100.0 IU/ml].

Inhalant: G = grass pollen; H = hazel pollen; O=oak pollen.

Food: ct = carrot; po = potato; wh = wheat; hz = hazelnut; am = almond; wn = walnut; cl = celery; ap = apple; p=peanut, s=soybean.

Table S2. List of mutagenizing primers.

Mutation	Mutagenesis primer sequences (5′-3′)
sD27A/L30A	GCTTCAGGTGCAACCTTTGGTGCGAGCTTTGCGCCATCAAGGATAAAGGCCT
ansD27A/L30A	CCAAAGGTTGCACCTGAAGCTGT

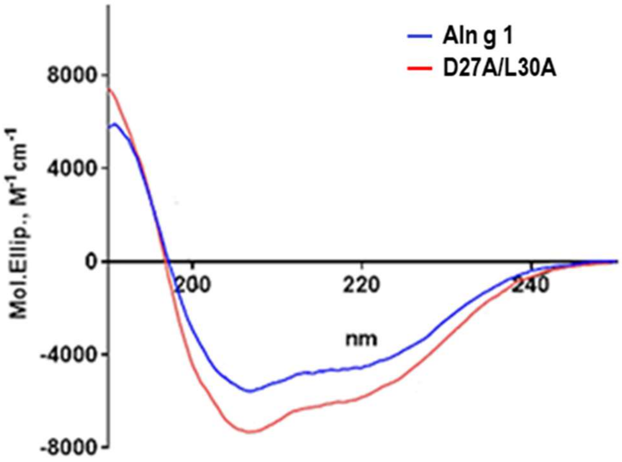

Figure S1. Overlay of the CD spectra of Aln g 1 and its mutant D27A/L30A measured at 25°C.
